# Knowledge, attitudes and practices regarding tuberculosis in a low-incidence area in the USA

**DOI:** 10.1099/acmi.0.001038.v3

**Published:** 2025-12-15

**Authors:** Jenna Randall, Nicole Kelp

**Affiliations:** 1 Department of Microbiology, Immunology, and Pathology, Colorado State University, Fort Collins, USA

**Keywords:** health communication, knowledge, attitudes and practices (KAP) survey, tuberculosis

## Abstract

Tuberculosis (TB) remains a large global health threat, including increasing cases in generally low-incidence areas of the USA. However, the knowledge, attitudes and practices (KAP) regarding TB in these low-incidence areas are underexplored, precluding planning for effective health communication in these areas regarding travel to high-incidence areas or potential future outbreaks in currently low-incidence areas. Using the health belief model as a theoretical framework, we developed a KAP survey to assess public perceptions of TB in Colorado, a currently low-incidence area. We collected complete responses from *n*=225 adults. We found that participants had higher self-assessed knowledge than actual knowledge about TB. We also found that while participants recognized TB as a global health threat, they were not personally worried about contracting TB. However, a portion of participants indicated that they would feel shame if they contracted TB. Public knowledge and risk perception about TB could be improved by providing information in low-incidence areas on the public health burden of TB. Additionally, providing health communication to focus on emotion management and reducing stigma about the disease would be important to promote healthcare-seeking and treatment compliance in case of a future outbreak.

## Data Summary

Anonymous, aggregate data are available in supplementary files.

## Introduction

Tuberculosis (TB) remains a large public health burden, with around 10.6 million people contracting TB globally in 2022. In total, 1.3 million of these cases resulted in death. In addition, public health and medical professionals remain concerned about antibiotic-resistant TB, with 410,000 cases of drug-resistant TB in 2022 (1).

Although these statistics show that TB is a significant global health problem, the disease burden is unevenly distributed. The WHO reports that 46% of cases were from South-East Asia, 23% were from Africa and 18% were from the Western Pacific. In contrast, North and South America only had 3.1% of the total cases [1]. In the USA, certain states have much higher incidence rates than others. Texas, California and New York, in particular, have a higher incidence of disease. In contrast, the Mountain West states, including Colorado, tend to have a very low incidence of disease [2]. However, a 2025 outbreak of TB in Kansas revealed the remaining threat of TB in the central and western USA and the need to study public perceptions about TB in this region [3].

In the field of public health, knowledge, attitudes and practices (KAP surveys) are often used in order to assess the knowledge of a population as well as the preconceptions or opinions that a certain group may have regarding a public health issue. This knowledge can be essential for guiding public health practices and programmes in a meaningful way [4].

KAP surveys can be built based upon different foundational behaviour theories, such as the health belief model (HBM) or the Risks, Attitudes, Norms, Abilities and Self-regulation (RANAS) framework. The HBM organizes how perceived threat (including perceived susceptibility and perceived severity), perceived benefits of doing a health behaviour to avoid the threat and perceived barriers to doing the health behaviour impact whether a person or group does a health behaviour, as well as what cues to action a person receives regarding the threat and behaviour [5]. In a KAP, attitudes relate to constructs like perceived threat, while practices relate to constructs like behaviours. RANAS is focused on identifying and targeting behavioural factors to change a subsequent behaviour [6]. Although this method can be useful for influencing community behaviours and guiding practices, we chose to employ the HBM model because we were not interested in changing behavioural factors but rather in identifying gaps in current KAP regarding TB. This initial study could identify potential targets for interventions using RANAS in the future.

Many previous studies assessed KAP surveys towards TB. However, these studies are often focused on TB patients in the USA [7], healthcare professionals [8,9] or the general public in a high TB incidence area [10,12]. These studies led us to the following research questions:

RQ1: What are gaps in public knowledge of TB in Colorado?

RQ2: What are Coloradoans’ perceived susceptibility and perceived severity towards TB?

RQ3: What are Coloradoans’ healthcare-seeking behaviours towards TB?

RQ4: How do past experiences with TB impact participants’ KAP towards TB?

RQ5: How does knowledge about TB impact attitudes?

This study was focused on the knowledge and attitudes of Colorado residents regarding TB as a model for low-incidence areas. This knowledge can help guide public health bulletins and teachings to improve knowledge of TB.

## Methods

### Survey design

See KAP survey questions in Tables1^4^ and Files S1 and S2, available in the online Supplementary Material. The knowledge questions began with participants self-rating their knowledge, followed by questions to assess participants’ actual knowledge. The questions focused on the basics of the disease of TB as well as its public health burden. These questions were guided primarily by the CDC’s website accessed in 2024 [2] as well as WHO resources.

**Table 1.** Demographics of participants

Category	Response option	No. of participants selecting each response (% of *n*=225 total participants)
Gender	Man	63 (25%)
	Woman	144 (64%)
	Non-binary	6 (2.7%)
	Other	0 (0%)
	Prefer not to say	5 (2.2%)
	No response	7 (3.1%)
Race/ethnicity	Asian	11 (4.9%)
	Black	2 (0.9%)
	Hispanic	9 (4%)
	Native American	5 (2.2%)
	White	184 (81.8%)
	Other	0 (0%)
	Prefer not to say	5 (2.2%)
	No response	9 (4%)
Highest education level	Some high school, no diploma	0 (0%)
	High school graduation or GED	6 (2.7%)
	Some college credit	26 (11.6%)
	Associate’s degree	24 (10.7%)
	Bachelor’s degree	87 (38.7%)
	Master’s degree	54 (24%)
	Professional/Doctoral degree	21 (9%)
	No response	7 (3%)

**Table 2.** KAP survey: knowledge – items, scoring and descriptive statistics for *n*=225 participants

Knowledge – self-assessed	Rate your knowledge about TB	1 (I know very little) to 5 (I know a lot)	1 to 5, Mean±sd	2.98±0.92
Knowledge – actual	How is TB contracted?	Sexual contact with an infected individual Contact with blood from an infected individual Eating infected food Sharing drinks/food with an infected individual From an infected individual coughing or sneezing From an infected mosquito	1 pt for ‘From an infected individual coughing or sneezing’ No pt for any other answer Mean±sd	0.86±0.34
	What infectious particle causes TB?	Virus Bacteria Fungi Parasite	1 pt for ‘Bacteria’ No pt for any other answer Mean±sd	0.61±0.49
	Which of the following are symptoms of TB? Check all that apply	Fever Chills Coughing Weight loss Nausea Haemoptysis (coughing up blood) Headache Chest pain Abdominal pain	0.25 pt for each correct symptom (Fever, Chills, Cough, Weight loss, Chest pain, Haemoptysis) Mean±sd	1.07±0.37
	Is TB treatable?	Yes No	0.5 pt for Yes 0 pt for No Mean±sd	0.42±0.18
	How long does a normal treatment course for TB take?	1 day 1 week 1 month 2–3 months 3–6 months 6–9 months 1 year	1 pt for 6–9 months No pt for any other answer Mean±sd	0.20±0.40
	How many TB cases were reported in Colorado in 2022?	0–10 10–1,000 1,000–5,000 5,000–10,000 10,000–1 million 1 million–10 million Over 10 million	1 pt for 10–1,000 cases No pt for any other answer Mean±sd	0.60±0.49
	How many TB cases were reported in the USA in 2022?	0–10 10–1,000 1,000–5,000 5,000–10,000 10,000–1 million 1 million–10 million Over 10 million	1 pt for 5,000–10,000 cases No pt for any other answer Mean±sd	0.32±0.47
	How many TB cases were reported worldwide in 2022?	0–10 10–1,000 1,000–5,000 5,000–10,000 10,000–1 million 1 million–10 million Over 10 million	1 pt for ‘Over 10 million’ No pt for any other answer Mean±sd	0.15±0.36

**Table 3.** KAP survey: attitudes – items, scoring and descriptive statistics for *n*=225 participants

Survey construct	Survey item	Response	Scoring (pt=point)	Score in our sample
Attitude – perceived severity	How serious is a TB infection if left untreated?	Scale from 1 to 5 with 1 being not serious at all and 5 being extremely serious	1–5 Mean±sd	4.5±0.72
Attitudes – worry and perceived susceptibility	TB is still a large global health concern	1–5 (Strongly disagree to strongly agree)	1–5 Mean±sd	3.98±0.96
	There is a potential I could become infected with TB in the future			3.08±1.06
	I worry often about getting a TB infection			1.60±0.85
Attitudes – perception of others with TB	I would avoid being around someone with a latent TB infection	1–5 (Strongly disagree to strongly agree)	1–5 Mean±sd	3.07±1.20
	I would avoid being around someone with an active TB infection			4.39±0.87
	If someone has a TB infection, it is their fault			1.41±0.49
Attitudes – emotions	If you contracted TB, which of these emotions would you feel? Select all that apply	Shame Fear Shock/surprise Sadness Stress Other	Number of participants selecting	21 142 56 2 14 1

**Table 4.** KAP survey: practices – items, scoring and descriptive statistics for *n*=225 participants

Category	Survey item	Response option	No. of participants selecting each response
Practices – healthcare-seeking and barriers	If you developed symptoms consistent with TB, at what point would you go to a healthcare professional?	Immediately after developing symptoms	69
		Within a week of developing symptoms	94
		Between 1 and 3 weeks after developing symptoms	46
		More than 3 weeks after developing symptoms	8
		I would not go to a healthcare professional	1
	What would prevent you from seeking medical advice if you were concerned that you may have contracted TB? Select all that apply	I can't afford it	25
		Lack of access to healthcare professionals	10
		Fear about contracting TB	1
		Embarrassment/shame about contracting TB	0
		Nothing would prevent me from seeking medical advice	136
		Other (please specify)	0
Cues to action	How often do you hear about TB in your daily life?	1 (very infrequently) to 5 (very frequently)	Mean±sd: 1.79±0.98
	Where in your daily life do you hear news about TB? Check all that apply	Television	19
		Newspapers	3
		Friends/family	9
		Social media	17
		Books	7
		Other (please specify)	39 (most common answer was workplace)
		I never hear news about TB	64
Past experiences	I have had a skin TB test	Yes	121
		No	61
		I don’t know	33
	I have had the BCG vaccine to help prevent TB (Note: this is not a part of a normal vaccine schedule in the USA)	Yes	27
		No	136
		I don’t know	54
	I know someone who has been infected with TB	Yes	39
		No	164
		I don’t know	14
	I have been infected with TB	Yes	39
		No	164
		I don’t know	14

The survey questions about attitude explored participants’ perceived susceptibility and severity and worry regarding TB as well as their emotions towards TB patients. The question regarding what emotions a participant would feel if they contracted TB was from Vericat-Ferrer’s KAP survey in Equatorial Guinea [13].

Finally, the practice questions focused on what actions participants would take to prevent TB or treat suspected TB infection, including what barriers would impede them from seeking treatment.

Participants also answered several background questions. These included demographic questions such as age, gender, ethnicity and education level and more specific questions about their experience with TB. To investigate whether previous TB experiences led to higher knowledge rates or unique attitudes about TB, participants selected whether they had previously had a skin TB test (PPD), the BCG vaccine or knew someone who was infected with TB. Finally, participants were asked how often they heard about TB in their daily life and from where (television, newspapers, etc.) to evaluate how much exposure an average Coloradan has to TB knowledge and the cues to action they had encountered.

### Survey distribution

Survey recruitment occurred in early 2024. Study participants were asked to confirm they were over 18 and a resident of Colorado. Participants were recruited through social media and flyers with QR codes placed in community areas, and the survey was completed online. This study was determined to be exempt by the Institutional Review Board of Colorado State University, and all participants consented to their survey responses being used for research.

### Data analysis

We scored the KAP survey based on the scoring system from a dengue KAP study, in which correct answers are given positive scores and incorrect or missing answers are given a score of zero [14] (see Table 2). Survey data were analysed using descriptive statistics as well as t-tests and linear regressions.

## Results

### Sampling

There were a total of 225 participants that completed the survey (see Table 1). Out of the participants, the majority were White (81%), with 64% respondents identifying as female, 20% identifying as men and 2.6% identifying as non-binary. Over 70% of participants had a bachelor’s degree or higher. Colorado’s demographics indicate that 62% of people are White and 28% of adults hold a bachelor’s degree or higher. Thus, our sample was more educated than the average Colorado populace, likely due to self-selection of those who chose to take a survey about TB. This suggests the need to further engage with Coloradans with lower degrees of education in terms of their knowledge about infectious disease. In addition, this survey may not reflect the attitudes and knowledge of non-White Coloradoans regarding TB.

### KAP survey descriptive statistics

Tables2^4^ list survey items, scoring process and descriptive statistics (mean±sd or number of participants selected for each response) for the survey.

### Knowledge

To answer RQ1 about participants’ knowledge gaps about TB, we assessed participants’ self-assessed and actual knowledge about TB. Participants’ mean self-assessed knowledge score was 2.98/5, with 47.9% of participants responding with a score of 3. For actual overall knowledge, participant responses were scored as listed in Table 2. Participants’ mean actual knowledge was 4.25/8. Linear regression showed that while the slope of correlation between perceived and actual knowledge was positive, this correlation was not significant (*P*=0.53). Individually, the difference between perceived knowledge percentage and actual knowledge percentage varied by an average of 6%, with people perceiving their knowledge as slightly higher than the actual knowledge.

Regarding specific actual knowledge questions, the majority of participants (86.7%) correctly selected coughs/sneezes as the mode of transmission for TB. However, around 35% of participants incorrectly selected sharing food/drinks as a mode of transmission for TB.

For the pathogen causing TB, although 62% of participants selected bacteria as the infectious particle responsible, 35.2% of participants believed it to be a virus. This has implications for participants’ knowledge of treatment options and antibiotics.

**Fig. 1.**
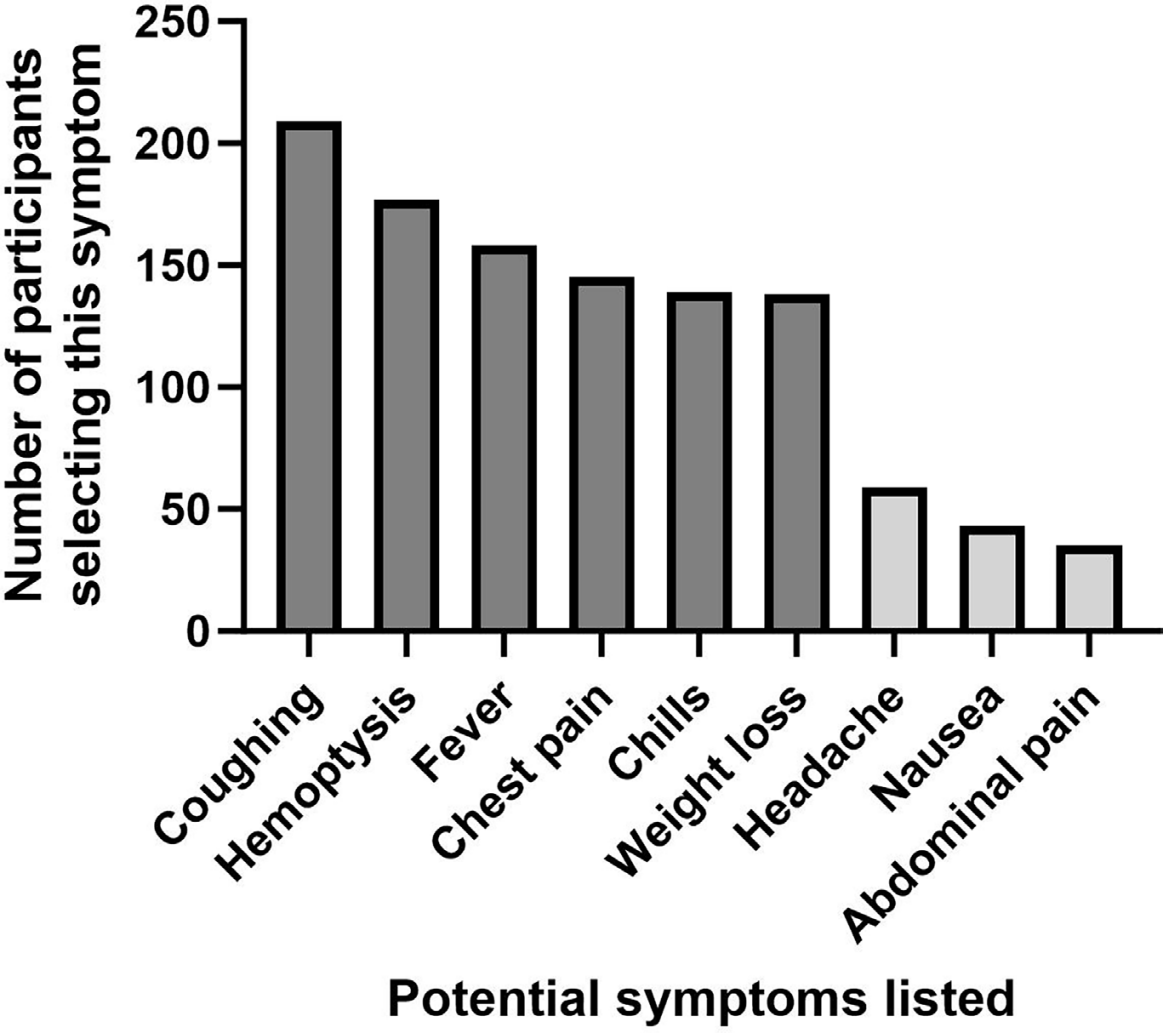
A bar graph displaying the number of participants that chose each symptom as a symptom of active TB. The correct symptoms given by the CDC are indicated in dark grey and the incorrect symptoms in light grey. The correct symptoms given by the CDC are listed as coughing, haemoptysis, fever, chest pain, chills and weight loss. Briefly, ~150–200 of the participants selected each of these symptoms. Incorrect symptoms are listed as headache, nausea and abdominal pain. Fewer than 50 participants chose each of these symptoms.

As shown in Fig. 1, participants were very accurate in selecting correct symptoms, with correct symptoms selected being selected more than 2.3 times more often than incorrect symptoms.

The final questions of the knowledge portion of the survey assessed participants’ knowledge of TB burden. Although 61.7% of participants and 32.8% of participants correctly identified the burden of disease in Colorado and the USA, respectively, only 15.3% of participants chose the correct answer for how many cases were reported worldwide.

### Attitudes

To assess participants’ perceived severity of TB (RQ2), we asked participants to grade the severity of an untreated TB infection (see Table 3). The average score for this question was a 4.5/5, indicating that the general Colorado public recognizes that TB can be an extremely serious disease. In total, 99% of participants selected a 3 or higher. Interestingly, while participants’ average perceived susceptibility to getting a TB infection in the future was 3.08/5, their average worry about getting a TB infection was 1.60/5, suggesting that generally participants are not worried about TB in their daily lives.

**Fig. 2.**
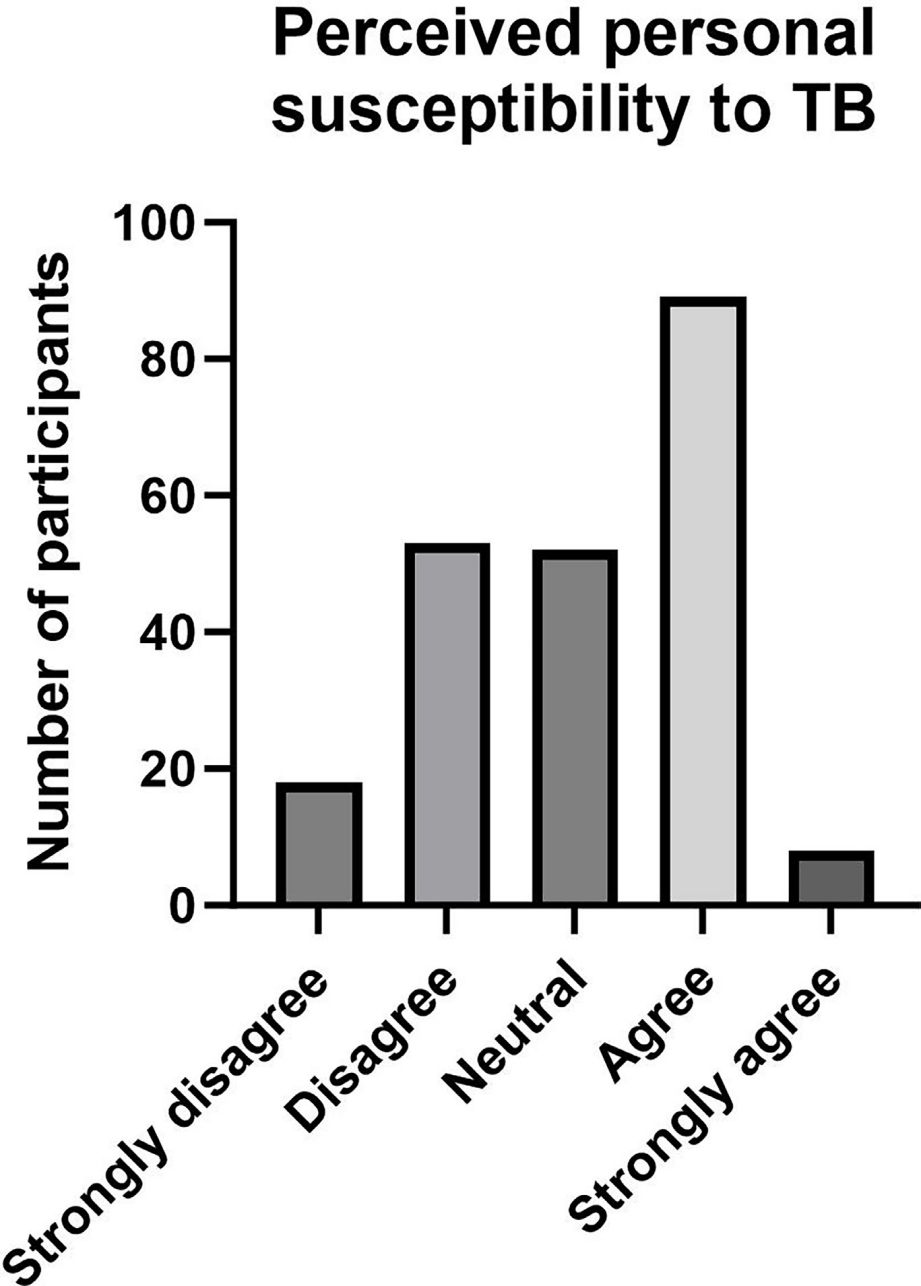
A bar graph displaying how participants ranked their personal susceptibility to TB. Briefly, ~60 participants disagreed or were neutral that they are personally susceptible to TB. Briefly, ~90 participants agreed that they are personally susceptible to TB. Few participants strongly disagreed or strongly agreed that they are personally susceptible to TB.

To assess participants’ perceived susceptibility to TB and overall worry for TB (RQ2), we assessed participants’ risk perception of TB in general. A large majority (78.4%) of participants agreed or strongly agreed that TB is still a global health concern. However, there was nearly a bimodal distribution on participants’ personal perceived susceptibility, with large numbers of participants selecting both ‘disagree’ and ‘agree’ in response to the statement ‘there is a potential I could become infected with TB in the future’, and very few participants strongly agreeing with personal susceptibility (Fig. 2).

The survey also asked two statements regarding how participants would react around someone with TB. Participants were neutral on whether they would avoid someone with a latent TB infection (average score 3.07/5) but agreed that they would avoid someone with an active TB infection (average score 4.39/5), suggesting that participants do understand the differences between latent and active TB.

Similarly, to gauge if there is a social stigma around TB patients, participants were asked whether they think someone’s TB infection is their fault. Only one participant selected ‘Agree’, with no participants selecting ‘Strongly agree’. However, participants were next asked to select all emotions they would feel if they were to contract TB. In this setting, 21 participants selected ‘Shame’, contradicting their earlier responses that it is not a patient’s fault for contracting TB. The other most common answers for this question were shock, stress and fear.

### Practices

We analysed both participants’ healthcare-seeking behaviours as well as their barriers to healthcare-seeking behaviours (RQ3) (see Table 4). The majority of participants (209 participants, 92%) indicated that they would seek healthcare between immediately and 3 weeks after developing symptoms. Lack of affordability was the most common barrier to healthcare indicated (25 participants, 10%), and zero participants indicated that shame about contracting TB would be a barrier to seeking healthcare. This is interesting given that ~10% of participants indicated that they would feel shame if they contracted TB; these results seem to suggest that participants may feel public shame or stigma but would not feel shame in front of a healthcare provider.

### Interaction of past experiences, KAP

To assess RQ4 regarding how past experiences with TB impacted knowledge, we performed an unpaired t-test between the knowledge scores of participants who had one or more past experiences with TB listed in Table 4 and those who did not. Of note, 140 participants indicated a past experience with TB. Those with experience had higher self-rated knowledge (mean=3.2/5) compared to those who did not have experience (mean=2.6/5), which was significantly different by unpaired t-test (*P*=0.00069). However, there was no significant difference in actual knowledge score between those who had past experience with TB and those who did not.

To assess RQ5 about how knowledge impacted attitudes, we built a multiple linear regression model with the following variables: self-assessed knowledge, actual knowledge, perceived severity, perceived susceptibility (average of three items related to perceived susceptibility in Table 3) and perception of others with TB (average of three items related to perception of others with TB in Table 3). The only significantly correlated variables were perceived severity and perception of others with TB. Thus, different attitudes about TB related to each other, but we did not detect an interaction between knowledge and attitudes about TB.

## Discussion

### Main finding of the study

Below we summarize the findings about each research question and the implications for health communication messaging.

For RQ1 (gaps in public knowledge of TB), we found that participants’ perceived knowledge score was higher than their actual knowledge score. This discrepancy between self-assessed and actual knowledge has also been shown for COVID [15] and other infectious diseases. Interestingly, a large majority of participants agreed that TB is still a large global health concern, but struggled to identify how many cases were reported in the USA and worldwide annually. Nearly all the participants selected a lower number of cases than the reported number. This shows that Coloradoans are not fully aware of the global health problem that TB presents. Finally, about a third of participants did not know that TB is caused by bacteria. This may have implications for both avoiding transmission and seeking treatment, as has been highlighted in other studies of TB KAP [13,16,20].

For RQ2 (perceived susceptibility and severity), we found that while more than half of our participants thought there was a potential they could be infected with TB in the future, most of the participants were not worried about contracting TB. A potential for this discrepancy is a mismatch between perceived susceptibility and perceived severity as per the HBM, as has been previously explored [21,22]. It is possible that participants assumed that with treatment, they do not have to worry about a potential TB infection. Other reasons for this discrepancy could be further explored via focus groups.

Interestingly, we found that while no participants strongly agreed that it is someone’s fault if they contract TB, 10% of participants indicated that they would personally feel shame if they contracted TB. Such mismatched stigma about infectious diseases has been found for sexually transmitted infections like HPV (human papillomavirus) [23,24]. While stigma towards TB is lower than towards sexually transmitted infections like HIV (human immunodeficiency virus) [25], it has been shown useful to explore the strategies for reducing HIV-associated stigma when working to reduce stigma associated with other infectious diseases like Zika and Ebola [26]. However, there is ‘a dearth of reliable information on the effectiveness of TB stigma-reduction interventions’ [27], so more work is necessary in this area. Interestingly, knowledge about infectious disease has been shown to not impact stigma (25), so exploring more emotion-related messaging strategies rather than focusing purely on knowledge and information transition could be useful [28,29].

For RQ3 (healthcare-seeking behaviours), we found that the majority of patients would eventually seek treatment if they experienced TB-associated symptoms. However, we noted that the most common barrier to seeking treatment is affordability. This issue has been well-documented for TB [30,31]. This suggests the importance of coupling health communication about TB with effective policies to reduce barriers to health behaviours like treatment [32].

For RQ4 (past experiences with TB), we found that those with past experience with TB had higher self-assessed knowledge, which can promote self-efficacy in health behaviours [33]. This suggests a unique health messaging strategy to utilize testimonials from those with TB experience to share with others in order to disseminate the knowledge and self-efficacy associated with these experiences. Such strategies of narrative testimonials have been explored for health communication campaigns about cancer [34], tobacco [35] and COVID vaccines [36]. Patient empowerment has been shown to be important in TB control [37], and patients and families sharing their stories to educate others could be a useful form of empowerment.

Finally, for RQ5 (impact of TB knowledge on attitudes), we did not find a correlation between knowledge and attitudes like perceived susceptibility or perceived severity in our population. This aligns with previous recognition that the traditional hierarchical behaviour change model in which knowledge precedes attitudes which influence behaviour does not always hold true [38]. However, we did find an interaction between some attitudes about TB. This suggests that health communication messages should focus on stories, narratives, emotions and guidance about feelings and attitudes, not just facts, to increase knowledge.

### Implications of the study

Previous KAP surveys of TB in high-incidence areas showed an average knowledge of TB and noted that younger participants and male participants seemed to know less [12]. A different study performed in Ethiopia noted that only about a quarter of participants knew that TB was caused by bacteria [11]. We found that although adults in a low-incidence area knew TB was a problem, many participants were unaware of the magnitude of case numbers in the USA and worldwide. More importantly, there were large percentages of participants who were unaware of TB transmission routes, symptoms, treatment times and what type of pathogen causes TB. Because early diagnosis and treatment are so essential for TB, improving knowledge of TB transmission and symptoms could decrease the time it takes for Coloradoans to seek medical attention and thus decrease negative outcomes from TB [39]. Although TB is not prevalent in Colorado specifically, case numbers are increasing in the USA [40]. This increase in TB rates, coupled with increasing travel and immigration rates, makes it more important that Coloradoans and other low-incidence-area residents are educated about TB. Further surveys and interviews should be done to evaluate how Coloradoans protect themselves from TB in order to better drive public health programmes.

### Limitations of this study

One of the limitations of this study is that it is primarily focused on the Colorado Front Range, which is more urban than rural. Thus, it is difficult to generalize about how the survey represents those who live outside the Front Range. In addition, the survey was sent out primarily on social media, which may have disproportionately influenced the sampling. Future comparisons between rural and urban Coloradoans or between those of different ethnic groups may reveal insights about the unique KAP of different communities.

## Supplementary material

10.1099/acmi.0.001038.v3 Uncited Supplementary Material 1.

10.1099/acmi.0.001038.v3 Uncited Supplementary Material 2.

## Abbreviations

HBM: health belief model
HIV: human immunodeficiency virus
KAP: knowledge, attitudes and practices
RANAS: Risks, Attitudes, Norms, Abilities and Self-regulation
TB: tuberculosis
