# Supplementary material for: Knowledge, attitudes and practices regarding tuberculosis in a low-incidence area in the USA
Source: Access Microbiol. 2025 Dec 15;7(12):001038.v3. doi: 10.1099/acmi.0.001038.v3 (PMC12721402; doi:10.1099/acmi.0.001038.v3)
Supplement: Uncited Supplementary Material 1. [file acmi-7-01038-s001.pdf]

Pre-Survey Self-assessment

1.) Rate your knowledge about Tuberculosis (TB)

1 (I know very little) to 5 (I know a lot)

Knowledge:

1.) How is TB contracted?

-Sexual contact with an infected individual

-Contact with blood from an infected individual

-Eating infected food

-Sharing drinks/food with an infected individual

- From an infected individual coughing or sneezing

-From an infected mosquito

2.) What infectious particle causes tuberculosis?

-Virus

-Bacteria

-Fungi

-Parasite

3.) Which of the following are symptoms of TB? Check all that apply.

-Fever

-Chills

-Coughing

-Weight loss

-Nausea

-Hemoptysis (coughing up blood)

-Headache

-Chest pain

-Abdominal pain

4.) Is TB treatable?

-Yes

-No

5.) How long does a normal treatment course for TB take?

-1 day

-1 week

-1 month

-2-3 months

-3-6 months

-6-9 months

-1 year

6.) How many TB cases were reported in Colorado in 2022?

-0-10

-10-1000

-1000-5,000

-5,000-10,000

-10,000-1 million

-1 million-10 million

-Over 10 million

7.) How many TB cases were reported in the USA in 2022?

-0-10

-10-1000

-1000-5,000

-5,000-10,000

-10,000-1 million

-1 million-10 million

-Over 10 million

8.) How many TB cases were reported worldwide in 2022?

-0-10

-10-1000

-1000-5,000

-5,000-10,000

-10,000-1 million

-1 million-10 million

-Over 10 million

<u>**Attitudes**</u>

1.) How serious is a TB infection if left untreated?

-1 (Not serious at all) to 5 (Extremely serious)

For Questions 2-7 please indicate whether you agree or disagree with the statement, on a scale of 1 (Strongly disagree) to 5 (Strongly agree).

2.) TB is still large global health concern.

3.) There is a potential I could become infected with TB in the future.

4.) I worry often about getting a TB infection.

5.) I would avoid being around someone with a latent TB infection.

6.) I would avoid being around someone with an active TB infection.

7.) If someone has a TB infection, it is their fault.

8.) If you contracted TB, which of these emotions would you feel? Select all that apply.

-Shame

-Fear

-Shock/surprise

-Sadness

-Stress

-Other

9.) How serious is a TB infection if left untreated?

For Questions 10-15 please indicate whether you agree or disagree with the statement, on a scale of 1 (Strongly disagree) to 5 (Strongly agree).

10.) TB is still a large global health concern.

11.) There is a potential I could become infected with TB in the future.

12.) I worry often about getting a TB infection.

13.) I would avoid being around someone with a latent TB infection.

14.) I would avoid being around someone with an active TB infection.

15.) If someone has a TB infection, it is their fault.

16.) If you contracted TB, which of these emotions would you feel? Select all that apply.

-Shame

-Fear

-Shock/surprise

-Sadness

-Stress

-Other

<u>**Practices**</u>

1.) If you developed symptoms consistent with TB, at what point would you go to a healthcare professional?

- Immediately after developing symptoms

- Within a week of developing symptoms

- Between 1 to 3 weeks after developing symptoms

- More than 3 weeks after developing symptoms

- I would not go to a healthcare professional

2.) What would prevent you from seeking medical advice if you were concerned that you may have contracted TB? Select all that apply.

- I can't afford it
- Lack of access to healthcare professionals
- Fear about contracting TB
- Embarrassment/Shame about contracting TB
- Nothing would prevent me from seeking medical advice
- Other (please specify)

3.) How often do you hear about TB in your daily life?

-1 (very infrequently) to 5 (very frequently)

4.) Where in your daily life do you hear news about TB? Check all that apply.

-Television

-Newspapers

-Friends/family

-Social media

-Books

-Other (please specify)

-I never hear news about TB

Past experiences

Please indicate "Yes," "No," or "I don't know" for each statement.

1.) I have had a skin TB test.

2.) I have had the BCG vaccine to help prevent TB (Note: this is not a part of a normal vaccine schedule in the US)

3.) I know someone who has been infected with TB.

4.) I have been infected with TB.

Demographics

1.) Age

2.) Gender

- Man
- Woman
- Non-binary
- Other
- Prefer not to say

3.) Race/Ethnicity

- Asian
- Black
- Hispanic
- Native American
- White
- Other
- Prefer not to say

4.) Highest education level

- Some high school, no diploma
- High school graduation or GED
- Some college credit
- Associates degree
- Bachelor’s degree
- Master’s degree
- Professional/Doctoral degree
